# Effect of propolis supplementation and breed on growth performance, immunity, blood parameters and cecal microbiota in growing rabbits

**DOI:** 10.5713/ab.21.0535

**Published:** 2022-04-29

**Authors:** Ibrahim Al-Homidan, Moataz Fathi, Magdy Abdelsalam, Tarek Ebeid, Osama Abou-Emera, Mohamed Mostafa, Mohamed Abd El-Razik, Mohamed Shehab-El-Deen

**Affiliations:** 1 Department of Animal Production and Breeding, College of Agriculture and Veterinary Medicine, Qassim University, Al-Qassim 51452, Saudi Arabia; 2 Department of Poultry Production, Faculty of Agriculture, Ain Shams University, Hadayek Shoubra 11241, Cairo, Egypt; 3 Department of Animal Production, Faculty of Agriculture, Alexandria University, El-Shatby, Alexandria 21545, Egypt; 4 Department of Poultry Production, Faculty of Agriculture, Kafrelsheikh University, Kafr El-Sheikh 33516, Egypt; 5 Animal Production Research Institute, Agriculture Research Center, Dokki 12618, Egypt; 6 Department of Food Science, Faculty of Agriculture, Ain Shams University, Hadayek Shoubra 11241, Cairo, Egypt; 7 Department of Animal Production, Faculty of Agriculture, Suez Canal University, Ismailia 41522, Egypt

**Keywords:** Cecal Microbiota, Immunity, Propolis, Rabbit Breed

## Abstract

**Objective:**

The present study was conducted to investigate the potential effects of dietary supplemented propolis in two growing rabbit breeds on growth performance, immune response, blood parameters, carcass characteristics, and cecal microflora composition.

**Methods:**

A total of 90 growing rabbits aged 6 weeks from two breeds (V-line and Jabali) were randomly allocated to 3 dietary propolis experimental treatments. The experimental treatments consisted of a 2×3 factorial arrangement with two rabbit breeds and three levels of dietary propolis supplementation (0, 250 mg/kg, and 500 mg/kg). Each sub-treatment has 15 rabbits. The experimental period lasted six weeks.

**Results:**

There were no significant differences in growth performance and carcass characteristics due to propolis administration. Propolis supplementation at a high level significantly increased (linear; p<0.05) cellular-mediated immunity compared with the unsupplemented group. Furthermore, the rabbits receiving propolis exhibited a significant increase (linear and quadratic; p<0.03) in IgM immunoglobulins compared to the control. The current study provides further evidence that the dietary inclusion of propolis can significantly reduce pathogenic bacterial colonization in growing rabbits. The total count of microflora, *E. coli*, and *Salmonella* spp. was significantly lower (linear; p<0.01) in supplemented rabbit groups compared to the control group according to the microbiological analysis of cecal digesta. Based on breed effect, the results indicated that Jabali rabbits (local) performed better than V-line rabbits (foreign) in the majority of the studied traits.

**Conclusion:**

Dietary propolis is promising for further investigation into improving intestinal health and enhancing immunity in growing rabbits.

## INTRODUCTION

Recently, utilizing natural materials in livestock nutrition instead of antibiotics is alternatively being developed. Due to adverse effects of antibiotics on both animal and human health, their use in animal feeding as a growth promoter has been entirely banned by the European Union since 2006. There are many natural feed additives used in animal and poultry feeding, e.g. probiotics, prebiotics, organic acids, phytogenic compounds, and zeolites [1–4]. Propolis receives considerable attention as a natural feed additive in animal nutrition. Propolis, bee glue, is a natural resinous substance collected by bees from plant buds and exudates. Bees use sticky propolis to protect their hive against various microorganisms (viruses, bacteria, and fungi). Flavonoids, aromatic acids, phenolic acids, terpenes, and phenolic constituents have been identified as principal components responsible for the biological and pharmacological activities of propolis samples [5–7]. Additionally, propolis appears to have antimicrobial [8–10], antioxidant [11], and anti-inflammatory [12,13] activities. In developed countries, consideration has been focused on the use of propolis as a health supplement suited for human beings [11,14].

Utilizing supplemented propolis in livestock production via feed or drinking water to improve productive performance and health status has been investigated [2,7,15,16]. Dietary supplementation of propolis has been confirmed to possess a favorable biological action on dressed carcass percentage and growth performance in growing rabbits [2,15]. Furthermore, using ethanolic extract of propolis in rabbits suffering from chronic diarrhea caused a decrease in the duration of diarrhea and improved feed intake and final body weight [16]. However, the mode of action of propolis is still not fully understood in rabbits. Thus, the objective of present study was to investigate the potential effects of dietary supplemented propolis in two growing rabbit breeds on growth performance, immune response, blood constituents, carcass characteristics, and cecal microbiota.

## MATERIALS AND METHODS

This experiment was carried out at the experimental rabbit farm at the College of Agriculture and Veterinary Medicine, Qassim University, Saudi Arabia in early spring season of 2019. The experiment started at 6 weeks of age and lasted 6 weeks. During the experimental period, minimum and maximum ambient temperatures were 13°C±0.6°C and 29°C± 0.7°C, respectively (mean±standard error). All experimental procedures, animal care, and handling were performed according to the animal care instructions of scientific research deanship and approved by the committee of health research ethics and animal care, Qassim University, Saudi Arabia.

### Husbandry, diets, and experimental design

A total of 90 healthy growing rabbits representing the Saudi local breed (Jabali, J) and an imported Spanish rabbit line (V-Line, V) were used in the current experiment. Forty-five rabbits aged 6 weeks of each breed were randomly distributed into three dietary propolis treatments (15 animals in each sub-group). Rabbits were housed individually in wire mesh cages (50 cm×40 cm×40 cm) equipped with feeding hoppers and drinking nipples. All the rabbits were kept under similar housing and management conditions. Propolis was administrated in feed in the concentration of 0, 250 mg/kg (low level), 500 mg/kg (high level). A commercial basal diet for growing rabbits containing 18.5% crude protein, and 9.4 MJ metabolizable energy/kg was used. Feed and water were available *ad libitum* to the animals.

### Growth performance

All rabbits were weighed at the beginning and end of the experiment. Body weight gain, feed intake, and feed conversion ratio (FCR) were determined for the overall experimental period (42 days). Data analysis was performed based on the individual records.

### Cell-mediated immunity

The *in vivo* response induced by injecting a mitogen was evaluated by injecting Phytohemagglutinin (PHA-P) into the left ear. At 12 weeks of age, 60 rabbits were randomly assigned (10 animals/subgroup) for cell-mediated immune response. Each animal was injected intradermally with 100 μg PHA-P (Sigma Chemical Co., St Louis, MO, USA) in 0.1 mL of sterile saline. Upon injection, the site of the needle was marked with permanent black ink to facilitate further measuring. The resultant swelling response in the ear was measured with a constant tension dial micrometer (Ames, Waltham, MA, USA) before injection and at 24, 48, and 72 h after PHA-P injection. Ear swelling was expressed as the difference between the thickness of the ear before and after injection.

### Humoral immune response

Haemagglutination assay was performed for assessing humoral immune response. Sheep red blood cells (SRBC), as foreign antigens were adopted to determine the total immunoglobulin in the blood. At the end of the eleventh week, the 10 rabbits per sub-group were assigned to challenge SRBC. Each rabbit was injected with 1 mL/kg body weight of 10% SRBC solution. One week later, blood sample was taken for determining antibodies formed against SRBC in the serum using 96-well plates. The antibody levels against SRBC were measured by a hemagglutination test using a 2% SRBC suspension. The serum was heat-inactivated at 56°C for 30 min and analyzed for total, mercaptoethanol (ME)- sensitive (presumably immunoglobulin M [IgM]) and ME-resistant (IgG) anti-SRBC antibodies, as previously mentioned in Fathi et al [17]. The antibody titer was expressed as the log2 of the reciprocal of the highest dilution giving a positive reaction.

### Blood biochemistry and oxidative profile

Approximately 5 mL of blood was obtained from each slaughtered rabbit into a heparinized tube. The blood samples were centrifuged (1,500×g for 12 min at 4°C) and the resulting plasma was collected and stored at −20°C for further analyses. The concentrations of total protein, albumin, cholesterol, and triglycerides were determined in the plasma using commercial kits (Biomerieux, Craponne, France). The difference between total protein and albumin concentrations was used to calculate globin concentration. Then, the albumin to globulin ratio was calculated. Total antioxidant capacity (TAC), the activity of malondialdehyde (MDA) and glutathione peroxidase (GSH-Px) activity were determined using commercial kits purchased from Biodiagnostic for diagnostic and research reagents, Dokki, Giza. Egypt, www.bio-diagnostic.com, as described by Fathi et al [3]. Total antioxidant capacity was measured depending on the ability of antioxidants to reduce hydrogen peroxide (H_2_O_2_). The determination was performed by the reaction of antioxidants in the blood sample with a defined amount of exogenously provided H_2_O_2_. The antioxidants in the sample eliminate a certain amount of the provided H_2_O_2_. The residual of H_2_O_2_ is quantified colorimetrically by an enzymatic reaction which involves the conversion of 3,5,dichloro-2-hydroxy benzensulphonate to a colored product. Lipid peroxidation was determined by the quantification of MDA levels. The level of MDA was determined from the MDA equivalence standard. Samples and standards were first reacted with thiobarbituric acid in acidic medium at high temperature (95°C) for 30 min to form a reactive pink product. Optical density was measured using an ultraviolet spectrophotometer at 532 nm against blanks prepared by using distilled water. Glutathione peroxidase was determined in erythrocytes. The red blood cells were collected from blood samples and washed with saline solution three times. Cold deionized water (4°C) was added to lyse cells. The resulting clarified supernatant was used in GSH-Px assay. Enzyme activity (reduction of organic peroxide) was spectrophotometrically monitored by decreasing absorbance at 340 nm.

### Slaughter and carcass characteristics

At the end of the experimental period, 10 rabbits from each sub-group (60 in total) were assigned for carcass evaluation. The rabbits were fasted for 12 h with free access to clean drinking water, and then sacrificed in the morning and weighed. After bleeding, they were dissected according to Blasco et al [18] and Fathi et al [19]. The slaughtered rabbits were skinned, and the hot carcasses were weighed and recorded. Organs including the liver, heart, kidney, spleen, and thymus gland were removed, trimmed, and weighed. Carcass parts (fore, mid, and hind parts) were weighed. All collected data was expressed as a percentage of live body weight. Cecum with its content was separated and weighed. The cecum length was measured in cm. The index for the spleen and thymus gland was calculated as a percentage of live body weight.

### Cecal microbial populations

Cecal contents of the same slaughtered rabbits were collected. The contents were carefully hand-stripped into sterile containers. Total aerobic bacteria, *E. coli* and *Salmonella* spp. were analysed in the cecal digesta according to the procedures described by McDonald et al [20] and Horn et al [21]. A sample (10 grams) from each treatment-group was aseptically taken and homogenized in 90 mL of sterile diluent (0.1% peptone water) using a stomacher (Model 400; Seward, West Sussex, England) for 30 seconds. Serial dilutions of digesta were prepared in buffered peptone water (1 g/L peptone, 8 g/L NaCl, and 0.5 g/L L-cysteine hydrochloride) for the enumeration of total aerobic bacteria and *E. coli*. Each dilution was cultured on selective media for each bacterial strain to be counted or detected. Nutrient agar was used for the total aerobic bacteria count, and MacConkey agar was used for the *E. coli* count. The culture plates for total aerobic bacteria and *E. coli* were incubated at 37°C in an aerobic environment. The colony-forming units (cfu) in log10 per gram for total aerobic bacteria and *E. coli* within digesta were counted based on the colony morphology and characteristics. For the detection of *Salmonella* spp., a sample (25 g) of cecal contents was pre-enriched in 225 mL of peptone water and incubated at 37°C for 16 to 24 h. For selective enrichment, 1 mL of peptone broth was transferred to 9 mL tetrathionate broth and incubated at 42°C for 24 h. From each selective enrichment broth, a 5-mm loop was streaked on selective plates of bismuth sulfite agar and incubated at 37°C for 24 h. *Salmonella* spp. was expressed as a percentage of detection.

### Statistical analysis

Two-way analysis of variance was performed using JMP software version 13.0 [22] with breed and propolis level as fixed effects. The statistical model is described as follows:


$${\text{Y}}_{\text{ijk}}=\mu +{\text{P}}_{\text{i}}+{\text{B}}_{\text{j}}+{\left(\text{PB}\right)}_{\text{ij}}+{\text{e}}_{\text{ijk}}$$

where: Y_ijk_ = the observation taken on the kth individual; μ = overall mean; P_i_ = the fixed effect of the jth propolis supplementation level; B_j_ = the fixed effect of the ith breed; (PB)_ij_ = interaction between breed and propolis supplementation level; e_ijk_ = random error assumed to be independent normally distributed with mean = 0 and variance = σ^2^.

All results were presented as mean, and the variability in the data was expressed as pooled standard error of the mean. The significance of difference among the groups was assessed using Tukey’s test. Statistical significance was considered when p<0.05. Polynomial contrasts and linearity were examined using regression procedures to describe the shape of the response to increasing concentrations of propolis supplementation and to determine the model of best fit, either linear or quadratic. The responses in optimal parameters to the propolis supplementation level can be modeled using the following quadratic equation:


$$\text{Y}=\text{a}+{\text{b}}_{1}\text{X}+{\text{b}}_{2}{\text{X}}_{2}+\text{e}$$

Where: Y = optimal response; a = intercept; b = coefficients of the quadratic equation; X = propolis level, and e = error.

## RESULTS

Table 1 summarizes the growth performance and feed intake of two rabbit breeds fed a diet supplemented with propolis. There were no significant differences in body weight between dietary treatment groups at the start or end of the experiment. Also, propolis supplementation did not significantly affect weight gain or feed intake traits. However, a slightly improvement in body weight gain was detected in low (4%) and high (8%) levels of propolis. A similar trend in FCR was observed in the low (9%) and high (7%) levels of propolis. When compared to V-line rabbits, Jabali rabbits had significantly higher body weight. In spite of the superiority recorded for body weight of Jabali breed, the body weight gain, feed intake and FCR did not differ significantly between the breeds. However, insignificant improvement in FCR was found in the V-line (3.2) compared with that of the Jabali breed (3.5). No significant interaction of propolis with the breed for growth performance and feed intake was recorded.

As shown in Table 2, dietary inclusion of propolis significantly increased (p<0.01) cell-mediated response in a linear manner in rabbits that received the high level as compared to rabbits received either no or low level of propolis after 48 or 72 h of PHA-P injection. This trend was found after 24 h of injection, but with no significance. The Jabali rabbits recorded higher cell mediated response, particularly at 24 and 48 after PHA-P injection compared to V-line rabbits. With regard to breed effect, the Jabali rabbits had a significant increase in cell mediated response compared with that of V-line counterparts at 24 and 48 h post-injection of PHA-P (p<0.05 and p<0.03, respectively). In terms of humoral immunity, rabbits receiving a low level of propolis significantly increased in quadratic manner the titre of total antibodies formed against SRBC compared with the control rabbits. Additionally, a significant increase (linear and quadratic) (p<0.03) for IgM antibodies was found in rabbits given either a low or high level of dietary propolis. Regarding IgY antibody titre, there was no significant difference among the groups given different propolis levels. Due to the breed effect, Jabali rabbits had significantly higher (p<0.01) IgY antibodies than V-line rabbits. An opposite significant tendency was noticed for IgM antibodies. No propolis by breed interaction was detected for either cell mediated or humoral immunity. Regarding the lymphoid organ index, there was no significant difference due to propolis supplementation in the index of spleen and thymus. However, Jabali rabbits exhibited a heavier relative weight of spleen compared to their V-line counterparts.

The results of the blood parameters and antioxidant status are summarized in Table 3. Overall, there were no statistically significant differences (p>0.05) among dietary treatments regarding blood parameters and antioxidant status. In terms of breed effect, no differences in the blood parameters were detected between the two breeds. However, Jabali rabbits had a significantly lower (p<0.02) cholesterol level compared with that of V-line counterparts. The same tendency was found for triglycerides level, but with no significant difference. A significant increase (p<0.05) in TAC was recorded in the V-line breed compared to the Jabali one. On the other hand, GSH-Px activity significantly increased (p<0.04) in Jabali rabbits compared with their V-line counterparts. Besides, the level of MDA was decreased in Jabali (2.1 nmol/mL) compared with V-line (3.1 nmol/mL), but this decrease was insignificant.

As shown in Table 4, the dietary inclusion of propolis did not affect the dressing percentage or the carcass yield of the fore, mid, and hind-parts. A significant increase in carcass weight, dressing percentage, fore-part, and hind-part percentage was associated with Jabali rabbits compared to V-line ones. Generally, no significant differences due to propolis by breed interaction were detected. Neither propolis supplementation nor breed did not influence internal organs except for the relative weight of the kidney. Jabali rabbits exhibited a significantly higher (p<0.01) percentage of kidneys compared with that of V-line rabbits.

The results of morphological description and microbial counts of caecum as affected by the addition of different levels of propolis in the diets of rabbit breeds are presented in Table 5. The weight and length of the caecum did not significantly respond to the increasing dietary level of propolis in all experimental rabbit groups. However, rabbits fed diets containing propolis showed a slightly higher relative weight of caecum compared with the rabbits that received a control diet. As detected from cecal microbial analysis, the total bacterial count was significantly reduced (p<0.01) in a linear response in rabbits receiving different levels of propolis. Similarly, supplementing propolis led to a linear (p<0.01) decrease in the *E. coli* of the caecum. Moreover, the results showed that feeding rabbits with diets supplemented with increasing levels of propolis linearly and quadratically (p<0.01) reduced the *Salmonella* spp. population in caecum content. Regarding the breed factor, there were no significant differences in caecum morphology (weight and length) due to rabbit breed. However, a significant decrease (p<0.01) in the relative weight of the caecum was found in the Jabali breed. Interestingly, the Jabali rabbits recorded significantly lower (p<0.01) bacterial count parameters compared with the V-line rabbits. Furthermore, the interaction between propolis level and breed type was highly significant (p<0.05 or p<0.01) for all bacterial count measurements.

## DISCUSSION

Propolis supplementation is one of the most important growth promoters used in farm animals and immuno-enhancer for human health. Based on the statistical analysis, there were no significant differences in either growth performance or feed efficiency due to propolis supplementation. Although the body weight gain was not statistically different among treatment groups, we found a numerically increase (4% and 8%) in groups receiving 250 mg/kg and 500 mg/kg propolis, respectively compared to those in the control group. This result is in harmony with the findings of Piza et al [23], who found that the inclusion of crude propolis at a level of 1.5% insignificantly increased the total weight gain at 75 days of age by about 162 g compared to unsupplemented New Zealand rabbits. No difference in body weight of New Zealand White rabbits fed a diet supplemented with ethanolic propolis extract was also found [24]. Similarly, no significant differences in performance and slaughtering traits of Japanese quail receiving 6 and 12 mL propolis ethanolic extract/kg diet were detected [25]. In addition, propolis supplementation at levels of 500 or 2,000 ppm did not significantly improve the performance (body weight, feed intake and feed conversion) in male broilers [26]. Nevertheless, a significant increase in body weight gain was recorded in V-line growing rabbits fed a diet supplemented with either 150 or 300 mg of propolis/kg [2]. Administration of propolis in combination with bee pollen significantly improved the growing performance of rabbits rather than using propolis alone [15]. In terms of feed utilization, Piza et al [23] reported that the inclusion of crude propolis up to 1.5% did not affect the feed efficiency or diet digestibility in New Zealand rabbits. However, there are a lot of discrepancy in the results of using propolis in animal feeding that might be related to the dosage administration, chemical composition, breed, age, and gender. Based on breed effect, the deterioration of body weight (initial and final) noticed in V-line as imported rabbits compared with Jabali as a native breed could be attributed to the deleterious effect of high environmental temperature during the experimental period. However, several studies conducted at the same farm animal station proved that the V-line breed did not adapt to prevailing environmental conditions [3,19,27]. Likewise, Iraqi et al [28] reported that a superiority in body weight and weight gain was recorded for the Egyptian Gabali breed compared to the V-line breed.

The results of cell mediated immune response revealed that propolis administration at a high level significantly increased the response to PHA-P injection in a linear manner compared to the other groups. The response of rabbits receiving a low level was intermediate. Several studies have shown that propolis supplementation activates the immune system in different animal species [7,29,30]. A significantly higher response to PHA-P injection in laying hens fed a diet supplemented with propolis compared with the control ones was found [29]. Similarly, Bayrami et al [30] reported that the ethanol extract of propolis inclusion increased immunity in rats. However, Attia et al [15] found that administration of propolis alone was unable to improve growing performance and immune response in rabbits, but an improvement occurred when propolis was administered in combination with bee pollen. In terms of humoral immune response against SRBC, it could be noticed that propolis addition significantly increased total immunoglobulins and IgM titer, particularly at low level compared to the unsupplemented group. An insignificant improvement was found for IgY in rabbits given a low propolis level. Many researchers have indicated that propolis supplementation increases macrophage activity and interleukin levels, which allow them to produce immunoglobulins [31–33]. Our results are consistent with those previously reported by Çetin et al [7], who observed that the inclusion of propolis at the level of 3 g/kg of diet (300 ppm) significantly increased serum IgG and IgM levels in laying hens. Similarly, Ziaran et al [34] reported that humoral immunity was modulated by different levels of propolis in the broiler’s diet. They observed that low levels of dietary propolis increased antibody titer, whereas high levels of propolis decreased antibody titer, thereby exhibiting a bell-shaped dose-response relationship. Furthermore, Taheri et al [35] observed a relatively negative effect of a higher concentration of propolis on humoral immunity in broilers, suggesting a crucial response to propolis dosage. Moreover, Scheller et al [36] indicated that the frequent increase in propolis administration or higher doses has an inhibitory effect on antibody production in mice. Cetin et al [7] stated that using 600 ppm propolis level did not produce a significant increase in IgG and IgM levels in blood serum compared to 300 ppm propolis level, attributing it to the high formation of benzene constituent associated with the higher level. They reported that benzene could have a negative effect on immune function. Ethanolic extract of propolis, when administrated in combination with formalized inactivated *Pasteurella multocida* vaccine, enhanced specific and nonspecific immune response and reduced mortality rate in rabbits [13]. In broiler chickens, propolis administration showed immunomodulation and decreased tissue damage caused by free radicals [37,38]. In addition, propolis has been found to suppress the adhesion of pathogens to the intestinal wall and, in turn, enhance systemic immune function, including the activity of natural killers and cytokine secretion [39]. In lymphoid organs, the current results indicated that the propolis administration had no negative influence on the index of spleen and thymus in rabbits. Concerning the breed effect, it was observed that the Jabali rabbits had a significant increase in cellular immunity, especially at earlier examined times (24 and 48 h) compared to V-line counterparts. The same trend was found in IgY of humoral immunity. This result is in agreement with findings obtained by El-Tarabany et al [40]. They found that the local breed of rabbits had superiority in most immunological parameters compared with exotic breeds. Consistent with our results, it has been reported that the Jabali rabbits recorded a significantly higher spleen index compared to that of imported New Zealand rabbits [41]. The positive effect of propolis administration on rabbit immunity could be attributed to increase antibody synthesis resulting from bigger lymphoid organs. In this context, Teo et al [42] reported that the increase in weight of immune organs correlated with enhanced proliferation of immune cells, which represented better immunity of the body in disease-free animals.

The results of blood biochemical analysis showed that propolis administration did not induce significant alterations in parameters. However, a numerical increase in globulin level (14% and 18%) was recorded in rabbits fed a diet supplemented with low or high levels of propolis, respectively compared to the control group. A significantly increase in blood globulin was found in rabbits fed a diet supplemented with 300 mg propolis as compared to the unsupplemented group [33]. A similar trend in rats was reported by Bayrami et al [30], who found a positive effect of propolis on blood globulin in a dose-dependent manner. Several studies have shown that propolis can provide an increase in immunoglobulin production [7,34]. Insignificant decrease in cholesterol concentration was found in rabbits fed a high propolis level compared with those given a low level or control groups (9% and 10%, respectively). A significant reduction in cholesterol level of rabbits receiving ethanolic extract propolis was found compared with their untreated counterparts [43]. The inclusion of propolis in rabbit feeding did not affect blood serum cholesterol [16]. Regard to breed effect, a significantly lower concentration of cholesterol was found in Jabali breed compared to V-line breed. Besides, an improvement in antioxidant profile was observed in local rabbit breed (Jabali) compared to imported-one (V-line). Significantly higher GSH-Pxand insignificantly lower MDA was recorded in Jabali rabbits, while a significantly higher level of TAC was recorded in V-line rabbits.

The dressing percentage and carcass parts, as an important economic issue for the rabbit meat industry, showed no difference due to propolis supplementation compared to the control group. Studies involving the use of propolis as a feed additive have shown consistent results in terms of carcass quality. However, the effects of dietary propolis supplementation on carcass yield of fattening rabbit were insignificant [2,15,24]. Additionally, our results showed that the relative weights of internal organs (liver, heart, and kidney) were influenced by dietary propolis inclusion. In agreement with the last result, Hashem et al [2] reported that the propolis administration (150 and 300 mg/kg diet) did not affect the relative weight of internal organs in growing rabbits. Furthermore, administration of propolis at 200 mg/kg BW alone or in combination with bee pollen did not affect carcass characteristics or relative weight of internal organs compared to the control [15,24]. Due to breed effect, the Jabali rabbits recorded a significant increase in carcass weight, dressing percentage, fore and hind parts compared to the V-line rabbits. These results agree with the previous reports conducted by Fathi et al [3,19].

Similar to the effect of dietary propolis on caecum morphology in our study, Coloni et al [24] showed that the caecum relative weight in growing NZW rabbits given a propolis alcohol extract was the same as in untreated rabbits. It is worthy to note that the inhibition effect of propolis supplementation on total bacterial count, *E. coli*, and *Salmonella* spp. was clearly observed. A significant reduction was recorded in all bacterial population parameters, and that effect was linearly dose-dependent. Moreover, *Salmonella* spp. does not exist in rabbits fed a diet containing a high level of propolis. Propolis has been reported to have antibacterial activity against a wide range of pathogens. An improvement in health status was noticed in rabbits suffering from diarrhea symptoms after supplying the drinking water with propolis for 10 days [16]. All propolis types, irrespective of origin and consequently the compounds they contain, have shown microbial activity [44]. Many studies suggest that propolis is characterized by great antibacterial properties against either gram-positive or gram-negative bacteria [10,45–47]. In addition, it has antiviral activity [48]. The present findings could be attributed to the beneficial effects of the biologically active components of propolis that participate in controlling and reducing pathogenic bacteria [49–51]. The anti-inflammatory effects of propolis have been attributed to its flavonoid, phenolic acid, and caffeic acid contents [52,53]. Propolis extract was found to have inhibitory effects on dihydrofolate reductase similar to non-steroidal anti-inflammatory drugs. Furthermore, flavonoids have been reported to inhibit the activity of enzymes involved in the conversion of membrane polyunsaturated fatty acids [52,54]. Besides, the presence of polyphenols and flavonoids is related to immunostimulatory action [55]. Caffeic acid was found to have an antioxidant effect and blocked the production of reactive oxygen species in neutrophils and xanthine/xanthine oxidase system [44]. Phenolics derived from propolis appear to protect the gastrointestinal tract by inhibiting the growth of pathogenic bacteria such as *Clostridium* spp, *Staphylococcus aureus* and *bacteriosides* spp. [39]. Regarding the breed effect, the Jabali rabbits had a good gut microflora compared to the V-line breed. This difference may enhance the immune response associated with the local breed (Jabali). Moreover, the interaction between level administration and breed was significant for bacterial count measures.

## CONCLUSION

The results revealed that the addition of propolis to rabbits’ diets has a beneficial effect in reducing the colonization of *Escherichia coli* and *Salmonella* spp. in cecum of treated rabbits. The improvement in cecal microbiota was pronounced in Jabali rabbits compared V-line counterparts. Propolis supplementation can greatly improve the immune function of growing rabbits via the beneficial modulation of cecal microflora. Meanwhile, the growth performance and carcass quality remained unaffected by the dietary inclusion of propolis. Although, propolis is suggested as an effective alternative feed additive in rabbit feeding to enhance immunity and health status, further research is recommended to examine high inclusion rates of propolis in rabbit diets for improving growth performance as well.

## Figures and Tables

**Table 1:** Effect of propolis level and breed on body weight, gain and feed conversion ratio in rabbits

Trait	Propolis level (P)			Breed (B)		SEM	p-value			
		
	Control	Low	High	V-line	Jabali		P		B	P×B

							Linear	Quadratic		
Initial weight (g)	1,005.5	989.1	984.7	895.3^b^	1,107.4^a^	46.5	0.85	0.96	0.02	0.92
Final weight (g)	2,111.9	2,140.6	2,184.9	2,067.1^b^	2,208.4^a^	34.1	0.37	0.92	0.03	0.74
Gain (g)	1,106.4	1,151.5	1,200.2	1,171.8	1,101.0	36.5	0.35	0.41	0.59	0.29
Feed intake (g)	3,870.8	3,675.5	3,913.4	3,762.6	3,891.5	84.6	0.83	0.26	0.51	0.58
FCR	3.49	3.19	3.26	3.21	3.53	0.20	0.14	0.26	0.24	0.64

SEM, standard error of the mean; FCR, feed conversion ratio

a,b Values in rows with different letters differ significantly.

**Table 2:** Effect of propolis level and breed on immune response and lymphoid organ index of growing rabbits

Trait	Propolis level (P)			Breed (B)		SEM	p-value			
		
	Control	Low	High	V-line	Jabali		P		B	P×B

							Linear	Quadratic		
Cell mediated immunity (swelling difference)^1)^										
After 24 h	0.29^b^	0.34^b^	0.40^a^	0.30^b^	0.38^a^	0.023	0.04	0.92	0.05	0.71
After 48 h	0.17^b^	0.21^b^	0.27^a^	0.19^b^	0.25^a^	0.016	0.01	0.82	0.03	0.49
After 72 h	0.12^b^	0.14^b^	0.19^a^	0.14	0.16	0.012	0.01	0.58	0.22	0.36
Humoral immunity (SRBC antibody titer)										
Total Ig	5.11^b^	6.21^a^	5.70^ab^	5.52	5.79	0.158	0.11	0.01	0.30	0.53
IgM	3.00^b^	3.68^a^	3.50^a^	3.72^a^	3.15^b^	0.098	0.03	0.03	<0.01	0.94
IgY	2.11	2.53	2.20	1.80^b^	2.64^a^	0.119	0.75	0.15	<0.01	0.28
Organ index^2)^										
Spleen	0.07	0.06	0.08	0.06^b^	0.08^a^	0.004	0.59	0.09	<0.01	0.32
Thymus	0.19	0.16	0.18	0.17	0.18	0.007	0.49	0.05	0.75	0.61

SEM, standard error of the mean; Ig, immunoglobulin.

1) Swelling difference =swelling differece at tested time – swelling differece at 0 time.

2) Organ index = (Organ weight)/(Live body weight)×100

a,b Values in rows with different letters differ significantly.

**Table 3:** Effect of propolis level and breed on blood biochemistry and antioxidant profile in rabbits

Trait	Propolis level (P)			Breed (B)		SEM	p-value			
		
	Control	Low	High	V-line	Jabali		P		B	P×B

							Linear	Quadratic		
Total protein (g/dL)	6.47	6.66	6.82	6.49	6.79	0.12	0.18	0.78	0.13	0.41
Albumin, (g/dL)	3.98	3.83	3.88	3.87	3.92	0.07	0.71	0.34	0.72	0.63
Globulin (g/dL)	2.49	2.84	2.94	2.62	2.87	0.11	0.21	0.48	0.20	0.52
Albumin/globulin ratio	1.89	1.45	1.46	1.54	1.63	0.10	0.12	0.22	0.78	0.49
Cholesterol (mg/dL)	75.95	74.84	68.30	77.9^a^	69.2^b^	3.10	0.24	0.92	0.02	0.13
Triglycerides (mg/dL)	85.16	79.68	83.90	84.1	82.1	2.83	0.95	0.55	0.77	0.12
TAC (mmol/L)	1.00	1.10	1.02	1.13^a^	0.95^b^	0.04	0.89	0.36	0.05	0.79
MDA (nmol/mL)	2.46	2.76	2.55	3.05	2.11	0.28	0.89	0.68	0.12	0.24
GSH-Px (U/mL)	37.71	32.57	36.64	32.5^b^	38.8^a^	1.62	0.70	0.75	0.04	0.11

SEM, standard error of the mean; TAC, total antioxidant capacity; MDA, malondialdehyde; GSH-Px, glutathione peroxidase.

a,b Values in rows with different letters differ significantly.

**Table 4:** Effect of propolis level and breed on carcass traits and relative weight of internal organs in rabbits

Trait	Propolis level (P)			Breed (B)		SEM	p-value			
		
	Control	Low	High	V-line	Jabali		P		B	P×B

							Linear	Quadratic		
Carcass weight (g)	1,141.5	1,057.4	1,122.5	1,027.7^b^	1,167.8^a^	30.21	0.79	0.25	0.02	0.96
Dressing (%)	51.4	51.7	51.1	49.7^b^	52.7^a^	0.41	0.78	0.59	<0.01	0.91
Fore part (%)	15.1	15.1	14.9	14.4^b^	15.5^a^	0.18	0.72	0.76	<0.01	0.77
Mid part (%)	15.14	15.64	15.70	15.23	15.69	0.29	0.43	0.72	0.38	0.67
Hind part (%)	21.17	20.96	20.49	20.04^b^	21.50^a^	0.21	0.20	0.77	<0.01	0.27
Liver	3.17	2.89	3.12	3.10	3.03	0.08	0.83	0.14	0.68	0.94
Heart	0.30	0.31	0.32	0.33	0.29	0.01	0.42	0.77	0.16	0.76
Kidney	0.77	0.73	0.73	0.70^b^	0.78^a^	0.01	0.29	0.59	0.01	0.72

SEM, standard error of the mean.

a,b Values in rows with different letters differ significantly.

**Table 5:** Effect of propolis and breed on cecal morphology and microbiota in rabbits

Trait	Propolis level (P)			Breed (B)		SEM	p-value			
		
	Control	Low	High	V-line	Jabali		P		B	P×B

							Linear	Quatratic		
Morphology										
Weight (g)	118.3	116.1	127.2	126.9	115.9	2.82	0.78	0.99	0.10	0.86
Length (cm)	46.3	45.8	45.3	46.4	45.3	0.67	0.55	0.99	0.37	0.68
Weight (%)	5.40	5.68	6.00	6.22^a^	5.31^b^	0.16	0.67	0.32	0.01	0.59
Bacterial count (log10cfu/g)										
Total	6.08^a^	5.45^b^	4.54^c^	5.61^a^	5.11^b^	0.10	<0.01	0.21	<0.01	0.05
*E. coli*	5.42^a^	4.36^b^	3.16^c^	4.49^a^	4.14^b^	0.14	<0.01	0.57	<0.01	<0.01
*Salmonella* spp.^1)^	80.00^a^	70.00^b^	0.00^c^	66.67^a^	33.33^b^	5.66	<0.01	<0.01	<0.01	<0.01

SEM, standard error of the mean.

1) Detection percentage. Zero *Salmonella* spp. stands for undetectable.

a–c Values in rows with different letters differ significantly (p<0.05).
